# The Effect of an Essential Amino Acid Supplement on Muscle Performance and Inflammation in an Animal Model of Overtraining

**DOI:** 10.3390/nu18162614

**Published:** 2026-08-10

**Authors:** Asher M. Harris, Tavor Ben-Zeev, Lior Binman, Chagai Levi, Inbal Weissman, David D. Church, Abby Fulbright, Arny A. Ferrando, Jay R. Hoffman

**Affiliations:** 1 Sport Science Program, School of Health Science, Ariel University, Ariel 40700, Israel; ash.harris135@gmail.com (A.M.H.); tavorbenzeev@gmail.com (T.B.-Z.); binmanlior@gmail.com (L.B.); c.ashraf5@gmail.com (C.L.); inbal1878@gmail.com (I.W.); 2 Center for Translational Research in Aging and Longevity, University of Arkansas for Medical Sciences, Little Rock, AR 72205, USA; dchurch@uams.edu (D.D.C.); afulbright@uams.edu (A.F.); aferrando@uams.edu (A.A.F.)

**Keywords:** fatigue, muscle physiology, exercise, stress, animals

## Abstract

Background/Objectives: Overtraining (OT) syndrome occurs when an athlete’s training load exceeds the athlete’s ability to recover, leading to a performance decrement. The use of nutritional interventions to increase resiliency to OT is a topic of interest for maximizing performance adaptations. The purpose of this study was to determine whether an essential amino acid (EAA) supplementation administered intraperitoneally and orally can mitigate performance decrements and inflammatory responses in an animal model of resistance training-induced overtraining. Methods: Thirty-two C57BL/6J mice were randomly assigned to four groups (n = 8 per group). One group served as a control (CTL), while the other three groups performed a 5-week resistance training ladder climb program. One group trained three days per week for the duration of the program (RT), while the training frequency of the other two groups doubled to six days per week to cause the OT stress. One group of animals were provided with EAA (RTOEAA), while the other received a sham (RTO). Changes (∆) in maximal carrying load (MCL) were assessed. Results: RTO experienced a greater decrease in ∆MCL than RT (*p* = 0.002) and RTOEAA (*p* = 0.029). No differences (*p* = 0.254) were noted between RT and RTOEAA. In addition, TNF-α expression within the plantaris muscle was significantly elevated in RTO compared to all other groups. Significant decreases in the androgen receptor were also noted for RTO in the soleus compared to RT and CTL. Conclusions: Results demonstrated that EAA supplementation was able to increase resiliency and reduce the inflammatory response to the volume overload stress.

## 1. Introduction

A fundamental principle for training is the principle of overload [1]. To induce physiological adaptations such as muscular hypertrophy, improved strength or increased endurance, the training stimulus must exceed what the physiological system is generally accustomed to [2]. As these adaptations occur, the improved level of performance requires the training prescription to be further adjusted to maintain the desired exercise prescription; this is termed progressive overload [1]. This generally occurs by increasing training load, training volume and/or a combination of both [1]. Although progressive overload is crucial for stimulating continued performance improvements, increasing the training stimulus without adequate recovery and balancing the progression of the overall stress applied to the body might decrease performance [3,4].

Inadequate recovery may result in significant performance decrements and can be part of a phenomenon known as the overtraining syndrome [4]. When this occurs the athlete’s performance is generally seen to decrease. The duration of this decrease can vary from a few days to several months in the more extreme cases. For this reason, overtraining is often referred to as a syndrome [5]. The physiological changes that occur during overtraining will affect the athlete’s ability to recover and to perform. Overtraining can be defined as a state of prolonged performance maladaptation in conjunction with physiological perturbations including endocrine disturbances, systemic inflammation, a decrease in libido, reduced immune response, and decreases in mood and decreased sleep quality [5,6]. Suppression of the immune system has been shown to increase susceptibility to infections, illnesses, and other immune-related issues [5,7]. Chronic and excessive training can result in an exaggerated inflammatory response contributing to chronic low-grade inflammation, which can adversely affect immune function [8]. In addition, increases in the inflammatory response may result in significant muscle degradations, leading to a reduction in neuromuscular function [6,9]. The overtraining syndrome has not only been reported in competitive athletes but is also regularly found among tactical athletes (e.g., soldiers) [10]. Soldiers, like highly motivated athletes, will often push themselves beyond their physiological limits. In a study examining military training and overreaching, Ikonen and colleagues [10] reported that plasma amino acid concentrations were significantly reduced during sustained military training. This is consistent with the results of recent research that has reported significant decreases in amino acid profiles in high-performance sprinters [11] and endurance athletes [12] during intense training cycles.

The use of dietary supplements may provide a non-pharmacological approach to increase resiliency to high-intensity, high-volume training that can increase the risk for overtraining. To the best of our knowledge, there are no previous studies that have examined the efficacy of EAA supplementation on increasing resiliency to overtraining stress in resistance-trained individuals. A previous study by Hoffman et al. [13] provided β-hydroxy-β-methyl butyrate (HMB), a metabolite of leucine, to combat soldiers during sustained field exercise and reported significant reductions in the inflammatory response and an ability to maintain skeletal tissue quality. This was supported by subsequent research that reported that a combination of calcium HMB and the probiotic bacillus coagulans GBI-30 attenuated the inflammatory response and maintained muscle integrity during intense military training [14]. Other investigations have reported increases in anti-inflammatory cytokine concentrations following β-alanine supplementation in special operation combat soldiers [15]. In light of these studies, the primary purpose of the present study was to examine the effect of EAA supplementation on enhancing resiliency to a volume-induced overtraining stress in mice. A second purpose was to examine the effect of the EAA supplement on attenuating the inflammatory response to the overtraining stress.

## 2. Materials and Methods

### 2.1. Experimental Design

**Figure 1:**
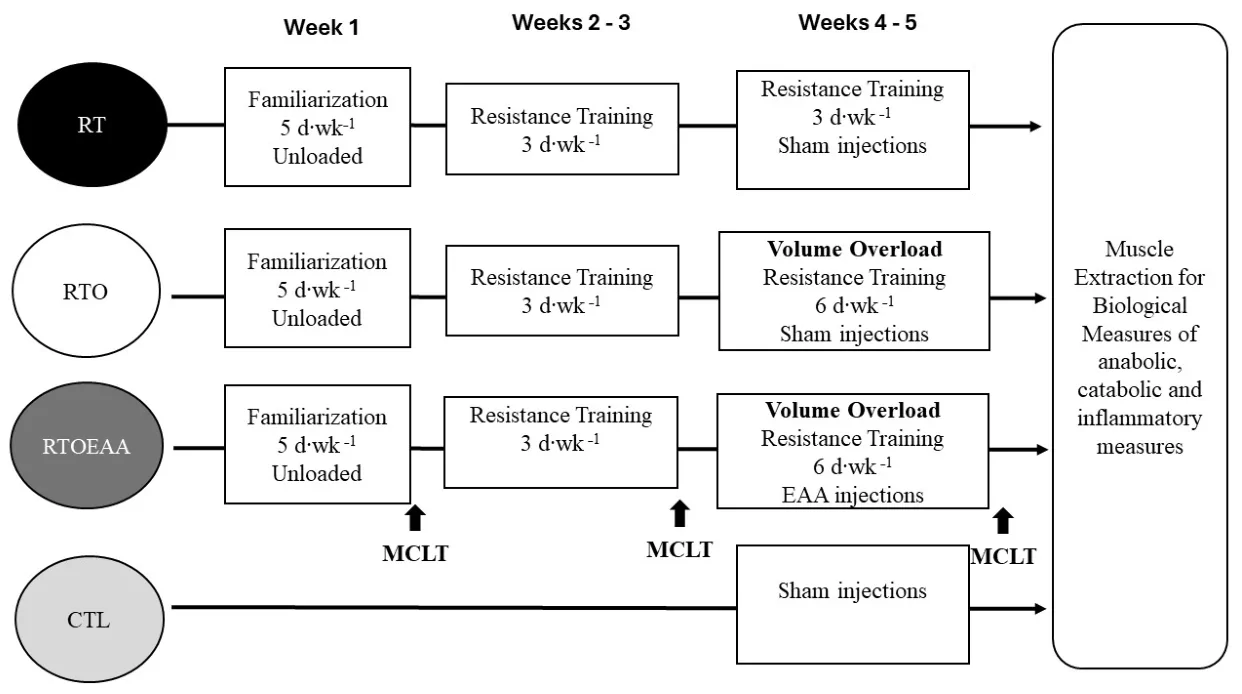
Study design. RT = resistance training; RTO = resistance training overtrained; RTOEAA = resistance training overtrained with essential amino acid supplementation; CTL = control; MLCT = maximum carrying load test.

Thirty-two C57Bl/6J 3-month-old mice (Envigo, Jerusalem, Israel), were acclimated to housing conditions for at least seven days. The mice were housed in groups of five per cage within a temperature-controlled vivarium set at 21 °C, with a reversed 12 h light/dark cycle. Food and water were provided ad libitum. Following acclimation, the mice were randomly assigned to one of four experimental groups (n = 8 per group): resistance training (RT), resistance training with overtraining (RTO), resistance training with overtraining and EAA administration (RTOEAA), or control (CTL). The control group was incorporated for biological marker comparisons and not performance comparisons. This is based upon previous research examining an animal marker of overtraining [16]. Investigators were not blinded to group allocation. The study design is depicted in Figure 1. This study was performed according to the principles and guidelines of the National Institute of Health Guide for the Care and Use of Laboratory Animals (Washington, DC, USA). All treatment and testing procedures were approved by the Animal Care Committee of Ariel University (AU-IL-2409-113, Ariel, Israel).

### 2.2. Resistance Exercise Protocol

For the three resistance training groups (RT, RTO and RTO-EAA), a ladder-climbing model was utilized as a resistance exercise. This model required the animals to climb a 1 m ladder at an 85° angle with 1.5 cm between each step [17]. The exercise protocol initially began without any resistance. This was considered the familiarization phase. During this phase of training, the mice climbed the ladder three times, starting from different positions (upper, middle, and base). Between each climb, the mice rested for 60 s. This protocol was performed for five consecutive days. Following the familiarization phase, a maximum load-carrying test (MCLT) was performed to assess the maximum carrying load (MCL) of a single ladder climb [18]. During the MCLT, the load was attached via a small bottle tied to the mouse’s tail. The initial starting weight was 10% of the mouse’s body mass and after every successful climb the load was increased by an additional 10% of the mouse’s body mass until failure. A 2 min rest period was provided between each attempt until failure. Failure was recognized as two consecutive unsuccessful attempts to climb the ladder. MCL was identified as the highest load carried.

During the initial two-week training period, ladder-based resistance training was conducted three times per week. During weeks four and five, both RTO and RTO-EAA engaged in six consecutive training sessions, whereas the RT group continued training three times per week. Each training session required the mice to perform eight sets of the ladder climb. A single climb was considered a set. For the first six sets, the mice performed two sets using a load equal to 50%, 75%, and 85% of their MCL. On the 7th set the mice completed one set at 100% of their MCL. If successful, 3 g was added to the mouse’s tail, and the mouse performed an additional climb (8th set). If not successful, the mice attempted the climb with the same weight. A two-minute rest period was provided between each climb. If the mouse was unable to complete the eight climbs successfully, the same resistance was maintained for the next training session. A logbook was maintained for all training sessions. This resistance training protocol was based upon previous work that demonstrated significant increases in muscle mass, muscle fiber cross-sectional area and maximum strength in mice using this protocol [19]. The volume overload in weeks four and five was designed to induce overtraining in the RTO and RTO-EAA groups. To ensure the overtraining groups completed a sufficient volume of training, the mice completed a total of eight sets. If a mouse was unable to complete a climb at the required load, the weight was reduced by 10% of their body mass (~3 g) until they could successfully complete the prescribed number of sets. Forty-eight hours following the last training session at week five, all the resistance-trained mice performed a final MLCT. No injuries or adverse events were observed during the training of the mice, and all mice completed all training sessions.

### 2.3. Determination of Overtraining

Although there is no established marker to determine overtraining, the overall consensus is that performance decrements are present [5,20]. As such, overtraining for this study was determined by the difference (delta) between the animal’s MLCT at the end of week three and the MCL of the final training session at week five. The difference provided a measure of the animals’ recovery. Poor recovery is another hallmark of overtraining [5,20].

### 2.4. EAA Administration Protocol

To ensure that the mice in the RTOEAA group would receive the desired daily EAA supplement intake (1.5 g · kg · day^−1^), intraperitoneal injections of EAA were provided for three consecutive days (days 4–6) during each week of the overtraining phase. EAA ingestion was also provided ad libitum in the animals drinking water at 2.5 g per 160 mL. The EAA supplementation was a proprietary blend provided by Amino Medical Science (Lewes, DE, USA) and contained leucine, isoleucine, valine, phenylalanine, lysine, threonine, methionine, and histidine and the non-essential amino acid tyrosine. All other groups received phosphate-buffered saline (PBS) injections at the same frequency.

### 2.5. Tissue Preparation

Twenty-four hours following the last assessment, mice were deeply anesthetized via an IP injection of 400 μL of 20 mg/mL Pentobarbital sodium (CTS Chemical Industries, Kiryat Malachi, Israel) was perfused transcardially with PBS. Plantaris and soleus muscles were extracted, snap-frozen in liquid nitrogen, and stored at −80 °C until analysis.

### 2.6. Western Blot Analysis

The plantaris and soleus muscles from each animal were homogenized using a manual homogenizer (Miulab Co., Ltd., Zhejiang, China) in ice-cold lysis buffer (RIPA; Merck KGaA, Darmstadt, Germany) and protease inhibitor cocktail 1% (Merck KGaA, Darmstadt, Germany). Protein lysates were obtained by collecting supernatants after removing insoluble materials by centrifugation at 8000 RPM (~7100× *g*) for 10 min at 4 °C. According to the manufacturer’s instructions, protein concentrations were evaluated using a BCA kit (Merck, Darmstadt, Germany). Samples were denatured by adding β-mercaptoethanol (Merck, Germany) mixed with sample buffer (Bio-rad, Hercules, CA, USA) at a ratio of 1:10 and heated at 70 °C for 10 min. The denatured protein content of the homogenates was separated using sodium dodecyl sulfate-polyacrylamide gel electrophoresis (SDS-PAGE). Equal amounts of protein (20 μg per lane) were loaded onto 4–20% Bis-Tris precast gels (MP42G12, Merck, Germany) and separated in MOPS SDS running buffer (MPMOPS, Merck, Germany) under constant current at 100 mA. In addition, a prestained protein ladder (Abcam, Waltham, MA USA) was loaded onto the gel to confirm the size of the proteins of interest. Separated proteins were transferred from the gel to a nitrocellulose membrane using midi transfer packs (1704159, Bio-rad, Hercules, CA, USA) and a turbo transfer device (Bio-rad, Hercules, CA, USA). After completion of the transfer, the membranes were washed with Ponceau S solution (Abcam, Cambridge, UK) to verify the completeness of the transfer. The membranes were then incubated in a blocking solution with 5% BSA diluted in PBS for 1 h at room temperature. Following the blocking incubation, membranes were incubated overnight at 4 °C with individual primary antibodies diluted in PBS containing 5% BSA, including mouse anti-glucocorticoid receptor (anti-GR, Santa Cruz biotechnology, Dallas, TX, USA, sc-393232, 1:400), rabbit anti-tumor necrosis factor-α (TNF-α, Abcam, ab-183218, 1:1000), and rabbit anti-androgen receptor (Anti-AR, Abcam, ab-108341, 1:1000). Protein levels were normalized as each membrane was incubated with mouse anti-α-tubulin (Santa Cruz, biotechnology, Dallas, TX, USA sc-5288) as the loading control. On the second day, the membranes were washed three times for 5 min with tris-buffered saline with Tween-20 (TTBS, Bio-Lab Ltd., Cat# 2089, Jerusalem, Israel) before being incubated for 1 h at room temperature in a cocktail of fluorescent secondary antibodies containing 5% BSA in PBS, anti-mouse Alexa Fluor^®^ 680 (Jackson ImmunoResearch, West Grove, PA, USA, 711–655-152, 1:15,000), and anti-rabbit Alexa Fluor^®^ 790 (Jackson ImmunoResearch, West Grove, PA, USA, 715–625-150, 1:15,000). The membrane was rewashed as described and scanned for protein visualization and analysis using the Odyssey CLx system (LI-COR, Lincoln, NE, USA) (resolution: 169 μm; intensity: auto mode), according to the manufacturer’s instructions. Mouse-derived antibodies (GR, α-tubulin) were detected in the 700 nm channel and rabbit-derived antibodies (AR, TNF-α) in the 800 nm channel. GR and α-tubulin were resolved by molecular weight (~95 and ~50 kDa) and assigned against the prestained ladder. Inspection of the individual channel images confirmed that each target was detected only in its expected channel. The fluorescence intensity measurements of the target protein bands of interest were determined using the Odyssey Infrared Imaging System software (Image Studio V5.2, Li-Cor Biosciences, Lincoln, NE, USA). All blot images are provided in the Supplementary Materials.

### 2.7. Statistical Analysis

For all performance and biological measures, a one-way analysis of variance (ANOVA) was used to compare performance results between RT, RTO and RTOEAA and for all molecular results between RT, RTO, RTOEAA and CTL. In the event of a significant F ratio, Tukey post hoc analysis was used for pairwise comparisons. Bivariate correlations between selected measures were performed using Pearson product moment correlations. Due to the novel nature of the animal model of fatigue used in this study, an a priori sample size calculation was not performed. All statistical analyses were analyzed using SPSS v29 software (SPSS Inc, Chicago, IL, USA), and an α level of *p* ≤ 0.05 was used to determine statistical significance. Effect size was determined if the comparison was significantly different using eta squared for ANOVAs and Cohen’s D for post hoc pairwise comparisons. Performance and body mass data was reported as mean ± SD, while all biological data was reported as mean ± SEM.

## 3. Results

**Figure 2:**
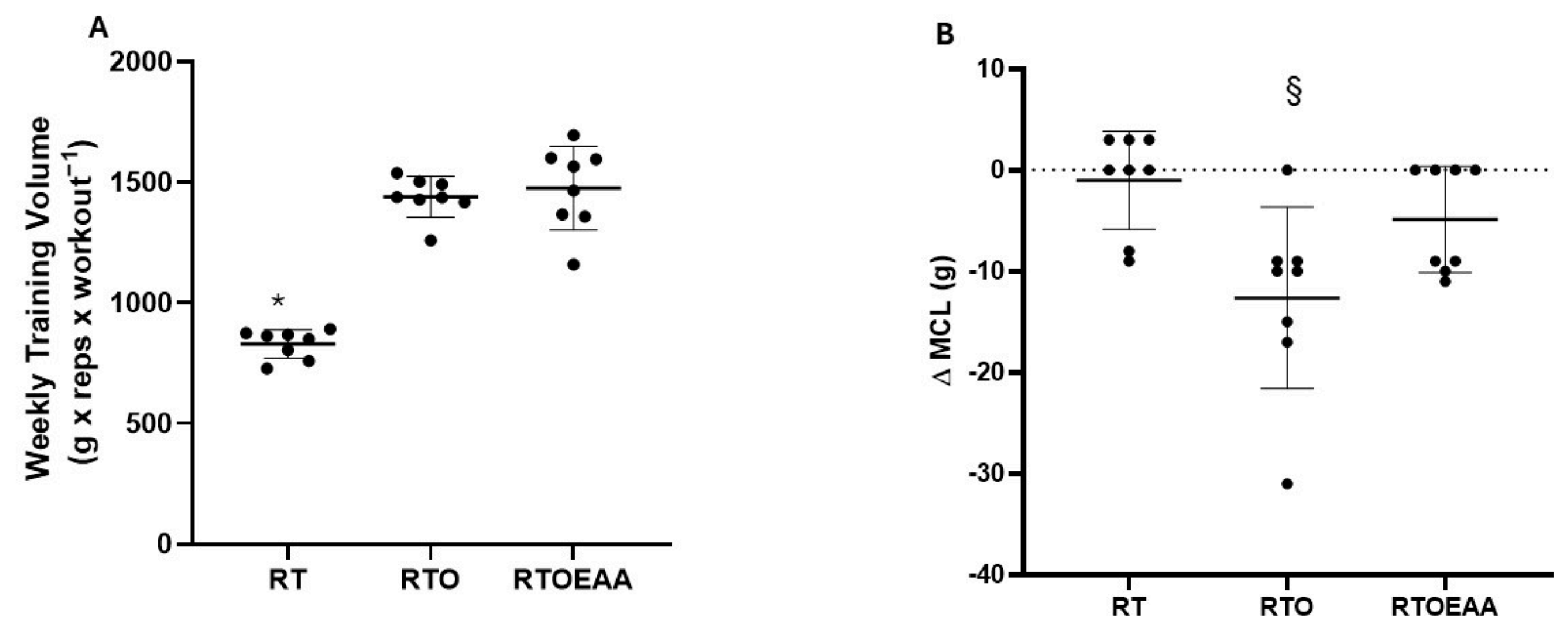
(**A**) Weekly training volume. (**B**) ∆ Maximal carrying load. RT = resistance training; RTO = resistance training overtrained; RTOEAA = resistance training overtrained with essential amino acid supplementation; § = RTO significantly lower than RT. All data are reported individual animal response and group mean ± SD.

The average weekly training volume in the three resistance training groups can be observed in Figure 2A. One-way ANOVA revealed a significant difference (F _(2, 21)_ = 77.13, *p* < 0.001, and η^2^ = 0.880) in the average weekly training volume during the five-week training protocol. The average weekly training volume for RTO and RTOEAA were significantly greater than RT (*p*’s < 0.001, respectively, d = 8.36 and 4.96, respectively), which reflected the doubling of the training days from three to six per week. Changes (∆ score) in MCL from the end of the third week of training to the end of the fifth week of training are depicted in Figure 2B. Results from the one-way ANOVA revealed a significant difference (F _(2, 21)_ = 6.407, *p* = 0.007, and η^2^ = 0.379) in ∆MCL. Significant differences were observed between RT and RTO (*p* = 0.006; d = 1.64). Although a trend towards a difference was noted between RTO and RTOEAA (*p* = 0.071), no other between-group differences were observed.

One-way ANOVA analysis of changes in body mass (week 5–week 1) revealed a significant difference (F _(3, 27)_ = 3.155, *p* = 0.041, and η^2^ = 0.260). Post hoc analysis indicated that the ∆ body mass for RTO (−0.56 ± 0.94 g) was significantly different (*p* = 0.005; d = 1.40) to CTL (0.63 ± 0.75 g) but not different compared to RT (−0.06 ± 0.94 g; *p* = 0.219) or RTOEAA (0.08 ± 0.34 g; *p* = 0.112). No other between-group differences were observed.

**Figure 3:**
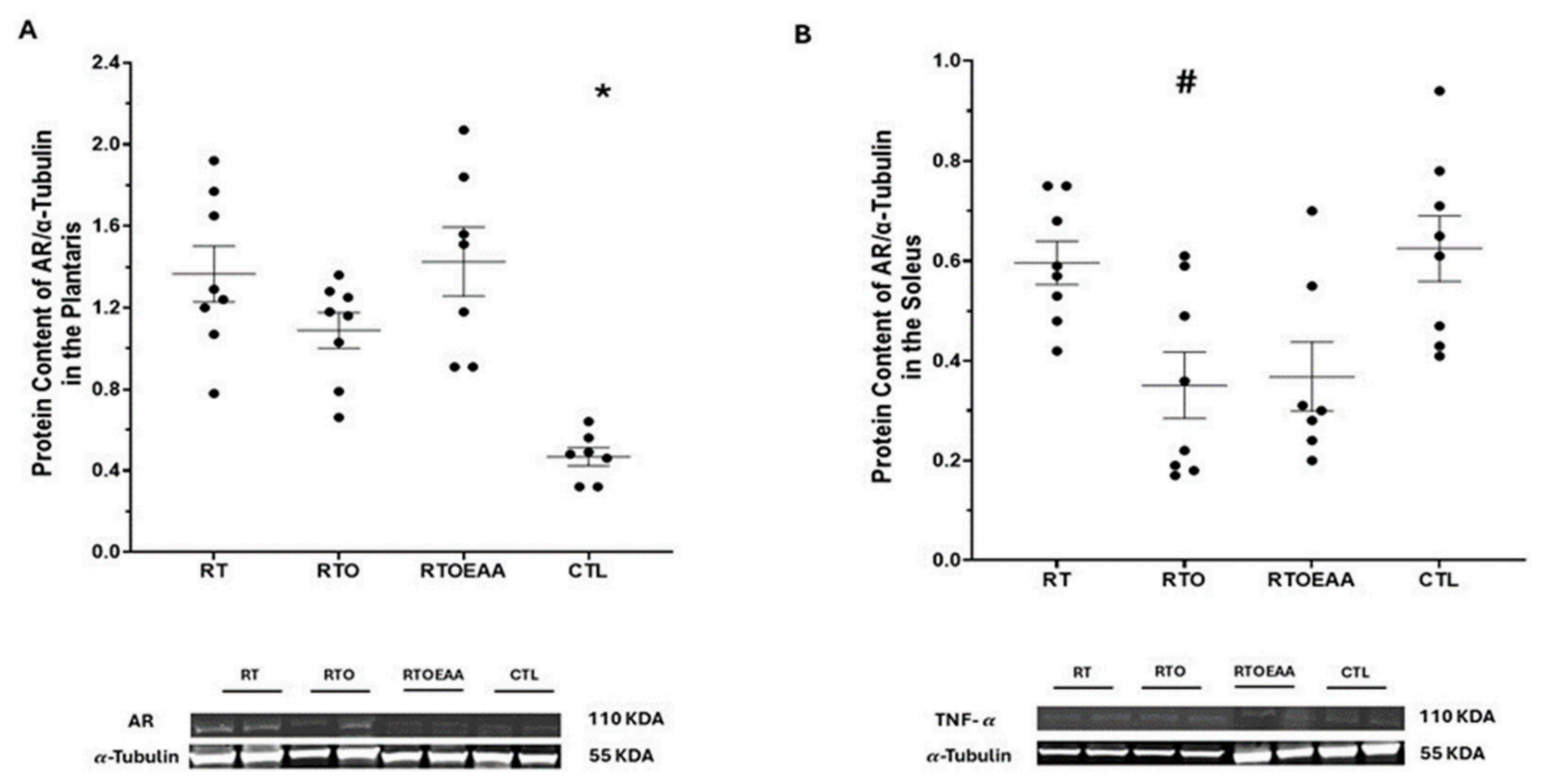
Androgen receptor (AR) expression for (**A**) plantaris and (**B**) soleus muscles. RT = resistance training; RTO = resistance training overtrained; RTOEAA = resistance training overtrained with essential amino acid supplementation; CTL = control; * = significantly lower than all other groups; # = significantly lower than RT and CTL. Representative Western blot images are shown below the quantified data. Individual animal responses and group mean ± SEM.

The effect of the study protocol on AR expression in the plantaris and soleus muscles can be observed in Figure 3A and Figure 3B, respectively. Significant differences in AR expression were noted in both the plantaris and soleus muscles (F _(3, 26)_ = 9.214, *p* < 0.001, and d = 0.515 and F _(3, 27)_ = 5.038, *p* = 0.007, and d = 0.359 respectively). Post hoc analysis revealed that AR expression for CTL in the plantaris was significantly lower than RT (*p* < 0.001; d = 10.31), RTO (*p* = 0.016; d = 10.35) and RTOEAA (*p* < 0.001; d = 2.08). No other between-group differences within the plantaris were observed. In the soleus muscle, AR expression for RTO was significantly lower than RT (*p* = 0.030; d = 1.40) and CTL (*p* = 0.048; d = 1.47). No other between-group differences in the soleus were noted. Although not significantly different, trends towards a difference were noted between RTOEAA and both RT (*p* = 0.053) and CTL (*p* = 0.080). No other between-group differences were noted. AR expression in the soleus was inversely correlated (r = −0.560; *p* = 0.005) to training volume.

**Figure 4:**
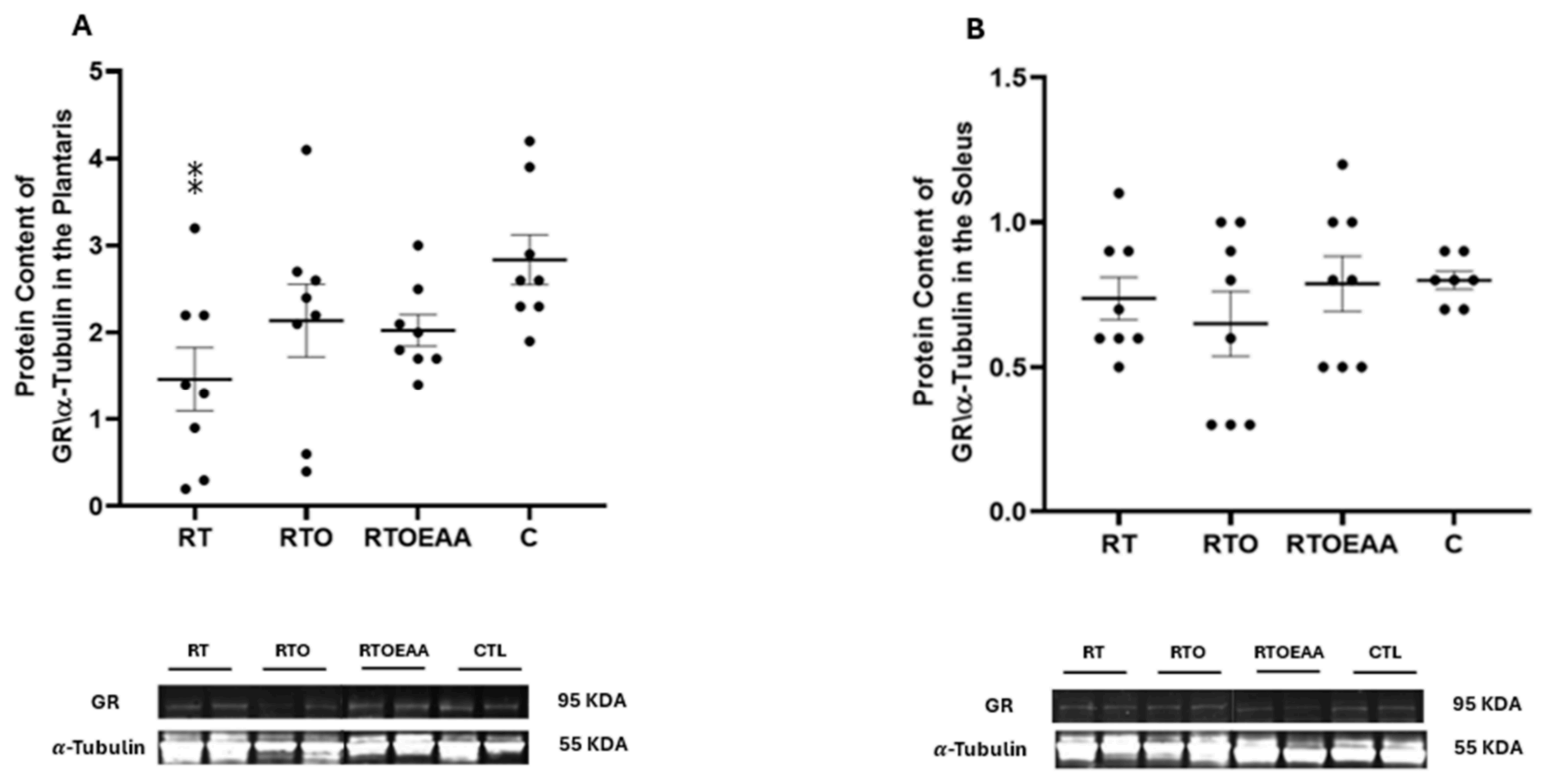
Glucocorticoid receptor (GR) expression for (**A**) plantaris and (**B**) soleus muscles. RT = resistance training; RTO = resistance training overtrained; RTOEAA = resistance training overtrained with essential amino acid supplementation; CTL = control; ⁑ = significantly lower than CTL. Representative Western blot images are shown below the quantified data. Individual animal responses and group mean ± SEM.

Changes in GR expression in the plantaris and soleus muscles are depicted in Figure 4A and Figure 4B, respectively. A significant difference in GR expression in the plantaris was noted (F _(3, 28)_ = 3.022, *p* = 0.046, and η^2^ = 0.245). GR expression in RT was significantly lower than CTL (*p* = 0.028; d = 1.49). No other significant differences were noted. In addition, no significant differences in GR expression within the soleus muscle was observed (F _(3, 27)_ = 0.628; *p* = 0.603).

**Figure 5:**
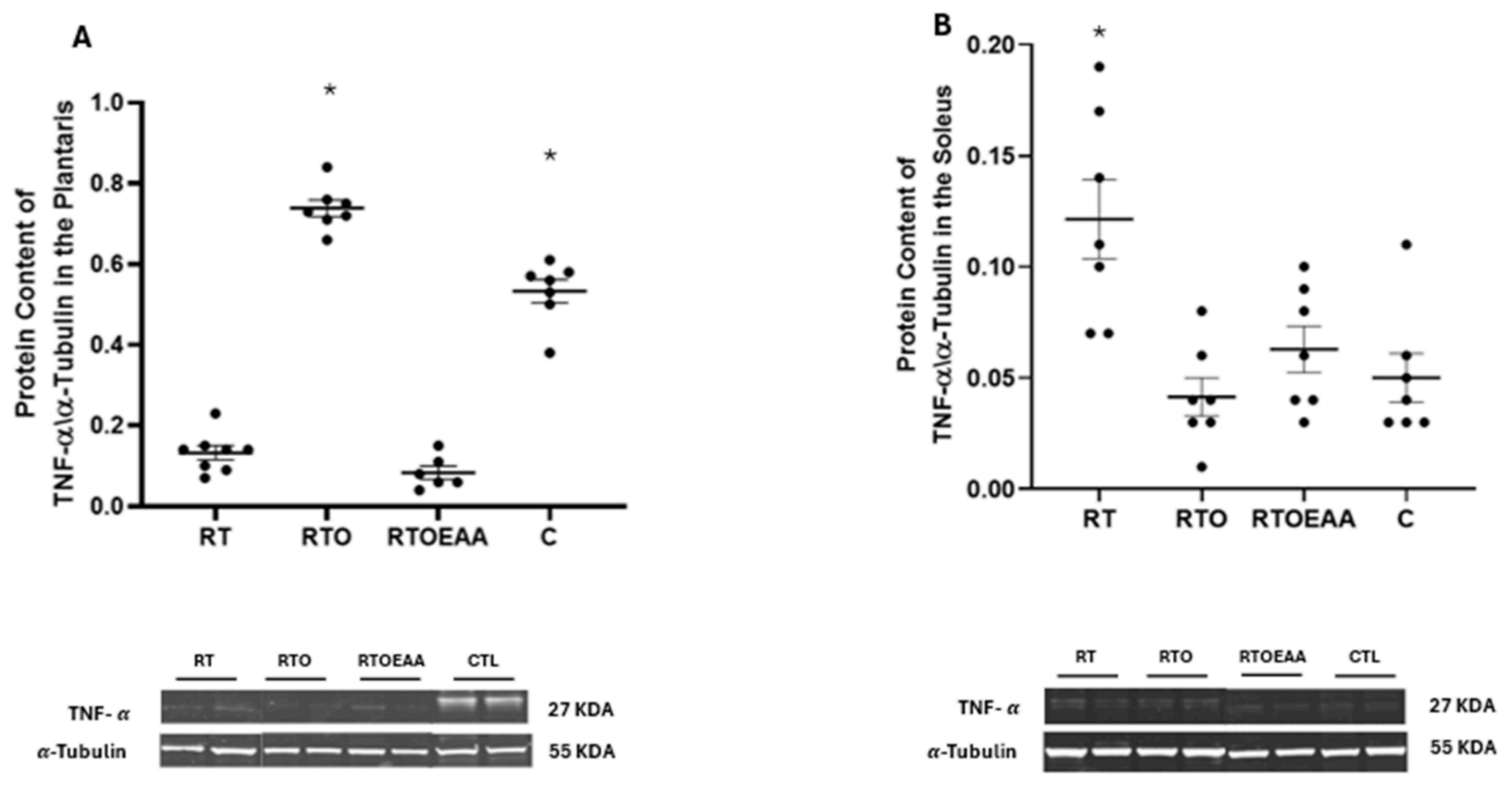
Tumor necrosis factor-α (TNF-α) expression for (**A**) plantaris and (**B**) soleus muscles. RT = resistance training; RTO = resistance training overtrained; RTOEAA = resistance training overtrained with essential amino acid supplementation; CTL = control; * = significantly different than all other groups. Representative Western blot images are shown below the quantified data. Individual animal responses and group mean ± SEM.

Comparison of TNF-α expression in the plantaris and soleus muscle can be observed in Figure 5A and Figure 5B, respectively. A significant difference in TNF-α expression was observed in the plantaris muscle (F _(3, 24)_ = 51.74, *p* < 0.001, and η^2^ = 0.861). Post hoc analysis indicated that TNF-α expression for RTO was significantly higher than RT (*p* < 0.001; d = 11.68), RTOEAA (*p* < 0.001; d = 13.37) and CTL (*p* = 0.004; d = 1.81). In addition, TNF-α expression for CTL was also significantly higher than RT (*p* < 0.001; d = 2.33) and RTOEAA (*p* < 0.001; d = 2.51). No other between-group differences were noted in the plantaris muscle. TNF-α expression in the plantaris was inversely correlated (r = −0.642; *p* = 0.002) to ∆MCL, as well as inversely correlated (r = −0.428; *p* = 0.023) to AR expression in the plantaris. A significant difference in TNF-α expression was also noted in the soleus muscle (F_(3, 24)_ = 8.839, *p* < 0.001, and η^2^ = 0.577). Post hoc analysis revealed that TNF-α expression for RT was significantly greater than RTO (*p* < 0.001; d = 2.23), RTOEAA (*p* = 0.13; d = 1.45) and CTL (*p* = 0.005; d = 1.82).

## 4. Discussion

The purpose of this study was to evaluate the efficacy of an EAA supplement on enhancing resiliency to a volume overload stress using an animal model of overtraining. Overtraining was induced by increasing the training frequency during weeks four and five, which significantly elevated the training volume in both the RTO and RTOEAA groups. The results of the study indicated that the animals in RTO were overtrained as their strength performance was significantly decreased compared to RT. This is consistent with the basic diagnostic criteria generally used to determine whether athletes are overtrained, which is a decrease in performance [4]. Furthermore, the EAA supplementation did appear to be efficacious in increasing the animals’ resiliency to the volume overload stress.

There have only been a limited number of studies that have examined the efficacy of EAA supplementation on increasing resiliency to overtraining. The scarcity of research examining the effect of a dietary supplement on overtraining in competitive or recreational athletes is likely related to the ethical consideration associated with causing athletes to become overtrained. However, the development of animal models of overtraining has also been limited, especially from a strength/power perspective. Recently, our laboratory reported that a resistance training animal model using volume overload stress can be used to cause significant decreases in performance, similar to what is observed during the overtraining syndrome [16,21]. One of those investigations demonstrated that EAA supplementation was effective in maintaining memory and neurotrophin expression in the hippocampus during the volume overload stress compared to animals that were not provided any supplement [21]. The only other investigation known that examined the effect of amino acid supplementation during volume overload stress was published more than 30 years previously [22]. In that study, an amino acid supplement containing both essential and non-essential amino acids was provided prior to meals and branched-chain amino acids (BCAAs) were provided prior to the daily workout in national-level weightlifters. A group of participating athletes were provided with a placebo and served as the control group. The week of volume overload resulted in significant decreases in jump height in both groups but no differences in strength, suggesting that the athletes may have been overreached but not necessarily overtrained. However, the short-term amino acid ingestion was unable to increase resiliency to the training stress. It is likely the relatively longer duration of training experienced by the animals (two weeks in an animal’s life versus a week in humans) likely resulted in greater physiological stress [23].

The basis behind the use of an amino acid supplement as a potential ergogenic aid to increase resiliency is based upon several studies reporting significant decreases in circulating amino acid concentrations in endurance-trained animals [24] and humans [25] who were overtrained. Decreases in amino acid expression were noted in both the brain and systemic circulation, which was associated with decreases in immune function. These studies and others led to the development of the BCAA hypothesis of overtraining that suggested that an amino acid imbalance resulting in changes in serotonin levels in the brain accounted for the increase in fatigue [26]. Despite the development of the hypothesis, the authors concluded that this theory was inconclusive as some studies showed promising effects [27,28], while others did not [29,30]. However, these investigations were primarily conducted performing exhausting endurance exercise with a focus on changes in brain function. Only limited research has been conducted on muscle function using a resistance training paradigm as the physiological stress causing overtraining. The physiological maladaptations associated with overtraining are different between endurance and strength/power athletes [31].

In addition to the performance measures, we examined anabolic, catabolic, and inflammatory biomarkers in both the plantaris and soleus muscles. Although high-intensity resistance exercise primarily activates fast-twitch fibers (e.g., plantaris) [32], the exercise protocol required the animals to climb a 1 m ladder, which took on average between 20 and 36 s as the animals progressed from low- to higher-intensity carrying loads. These relatively long duration repetitions could have potentially activated the slow-twitch muscle as well (e.g., soleus).

To ascertain changes in anabolism and catabolism within the muscle tissue, the expression of both androgen and glucocorticoid receptors were analyzed. Significant differences in AR expression in both the plantaris and soleus muscles were noted. However, the nature of the response differed between the muscles. In the plantaris, AR expression for CTL was significantly lower than all other groups. This is consistent with the anabolic response of muscle to a resistance training protocol [33]. In general, resistance exercise is a potent stimulator for AR upregulation. The volume overload stress did not result in any significant differences between RTO and RT or RTOEAA. However, AR expression for RTO was 24% and 23% lower (*p*’s > 0.05) than RT and RTOEAA, respectively. Similar AR expression following an overtraining protocol in mice was also noted by da Rocha and colleagues [34]. In their investigation, which used a downhill-running protocol for the stressor, no changes in AR expression were noted in the extensor digitorum longus muscle, a predominantly fast-twitch muscle. The only other study to examine the anabolic response to a volume overload stress in resistance training was conducted by Fry and colleagues [22]. In their study examining the effect of a volume overload stress in elite junior weightlifters, a significant decrease in resting testosterone levels was observed. However, when provided with an amino acid supplement, no differences in circulating testosterone concentrations were noted. The similar response in AR expression between RTO and RTOEAA observed in this study appear to be consistent with those results.

AR expression in the soleus muscle for RTO was significantly lower than RT and CTL groups. Although no significant differences were noted between RTOEAA and the other groups, trends towards a decrease were noted between RTOEAA and RT (*p* = 0.053) and RTOEAA and CTL (*p* = 0.080). No significant differences were noted between RTO and RTOEAA, providing additional support that the EAA supplement had minimal effect on changes in AR expression. These results were also consistent with what has been previously reported on changes in AR in an overtraining model. In a study examining downhill running in mice, a significant downregulation was noted in AR expression within the soleus [34]. However, that study utilized a running protocol, which would very likely preferentially recruit slow-twitch fibers. Nicoll and colleagues [35] also observed a downregulation of the AR following a volume overload stress in the vastus lateralis in experienced resistance-trained men. However, they did not examine whether the downregulation occurred in specific fiber types. The vastus lateralis is a muscle containing both fast-twitch and slow-twitch fibers. Thus, it is not possible to determine whether there was a preferential response between fiber types in that specific study. The results of the present study suggest that AR expression in the soleus muscle was more sensitive to the overload stress than AR within the plantaris muscle. This is supported by the significant inverse correlation observed between AR expression in the soleus and training volume. However, these results are in contrast with current knowledge regarding muscle fiber type sensitivity to training stress. Limited evidence suggests that the fast-twitch fibers are more sensitive to both mechanical and metabolic stress from exercise [36]. Additional research appears to be warranted examining muscle fiber sensitivity and the anabolic response to volume overload stress.

The volume overload stress did not appear to have any effect on GR expression in either the plantaris or soleus muscles. This contrasts with the work of others that observed a significant downregulation of GR in both the EDL and soleus muscles in mice performing both downhill and uphill training [34]. It is likely that differences in the mode of exercise used resulted in different metabolic and mechanical stresses that may result in a different molecular response within skeletal tissue. The significantly lower GR expression in the plantaris muscle observed in RT compared to CTL may represent a positive adaptation to the resistance training program. Previous research has demonstrated a downregulation of the GR from an aerobic training program in rodents [37]. However, another study investigating the effect of resistance exercise in previously untrained men reported an upregulation of the GR following a 12-week training program [38]. It was suggested that the upregulation of GR was the result of the metabolic stress associated with the training program and consistent with the elevations in circulating cortisol concentrations. Others examining volume overload resistance training stress in trained men reported no changes in GR expression in the vastus lateralis muscle [35]. It appears that changes in GR as a marker of overtraining is inconclusive.

The EAA supplement did not appear to have any influence on the GR response to the volume overload stress. Considering that no differences were noted in the GR response to the volume overload stress, the effect of the supplement was not surprising. There are only a limited number of studies that are known to have examined the effect of whole protein and amino acid supplementation on molecular changes within skeletal muscle. Nicoll and colleagues [39] reported no influence of a pre-workout supplement containing the non-essential amino acids glycine and taurine and the nutrients beta-alanine and creatine. No other studies are known that have examined the effect of an amino acid supplement containing EAA on changes in the glucocorticoid receptor response to volume overload stress.

TNF-α expression within the plantaris reflected the significant inflammatory response associated with the volume overload stress that RTO experienced. This is consistent with previous research demonstrating that overtraining will cause significant elevations in pro-inflammatory cytokines, including TNF-α [34,40]. Elevations in TNF-α expression within the plantaris was observed to be inversely correlated to ∆MCL, indicating that the greater the inflammatory response the greater the decrease in strength was observed. This is consistent with previous research that demonstrated a significant inverse relationship between circulating TNF-α concentrations and dynamic strength measures (e.g., leg press and leg extension) in older adults [41]. In addition, these results also support the understanding that TNF-α accelerates protein degradation within skeletal muscle [42]. More so, TNF-α was demonstrated to be inversely correlated to AR expression within the plantaris, providing further evidence of the deleterious effects of elevations in TNF-α expression has on anabolism within skeletal tissue. Differences in TNF-α expression in the soleus observed in this study was not consistent with current understanding and is difficult to explain. The results were most likely spurious in nature.

The EAA supplement attenuated the inflammatory response to the training stress as reflected by the significantly lower TNF-α expression in the plantaris for RTOEAA compared to RTO and CTL. This is consistent with previous studies that have demonstrated that EAA ingestion can reduce the inflammatory response to training stress in both animals [43] and humans [44,45]. These investigations, and the present study, used supplements containing EAA including leucine. Leucine has an important role in attenuating the inflammatory response by reducing the response of pro-inflammatory cytokines such as TNF-α, enhancing debris removal via macrophage activation coinciding with activation of anti-inflammatory cytokines and also enhancing muscle recovery and regeneration by stimulating muscle protein synthesis [46]. In addition, leucine also has an important role in stimulating mTOR expression, which regulates muscle protein synthesis [47]. Elevations in muscle protein synthesis could have been another mechanism that contributed to the improved performance in RTOEAA. Unfortunately, this was not assessed in the present study.

The study did not use an a priori sample size calculation to determine sample size due to the novelty of the investigation. Although statistical differences were noted, this is still a limitation and may have impacted some of the molecular measures examined. In addition, the EAA supplementation provided to the mice was a propriety blend; as such, we are not able to provide the percentage contribution of the specific amino acids that the supplement contains. This makes it difficult for subsequent investigators to duplicate our results. Finally, the combined IP and oral supplementation pattern is not a typical pattern used by athletes and should be interpreted accordingly.

## 5. Conclusions

The results of the study should be interpreted in the appropriate context in that it was conducted in a murine model and cannot be extrapolated to what would happen in a human study. Still, the results of this study support the use of an EAA supplement to increase resiliency to volume overload stress. The evidence presented demonstrated improved performance outcomes in animals that were supplemented with EAA that were similar to animals that trained without the volume overload. These data were supported by the molecular changes observed within the skeletal muscle. Significant elevations in the inflammatory response noted in RTO were contrasted with the attenuated response observed in animals that experienced the volume overload but were supplemented with EAA. Future research may wish to examine longer-duration EAA supplement protocols and a dose–response effect from EAA supplementation. Finally, future investigations will need to utilize specific human populations (e.g., competitive, or tactical athlete populations) for translational purposes.

## Data Availability

The data presented in this study are available on request from the corresponding author.

## Supplementary Materials

The following supporting information can be downloaded at https://www.mdpi.com/article/10.3390/nu18162614/s1, Supplementary Figure S1. α-tubulin Fluorescence Intensity. RT = Resistance training; RTO = Resistance training overtrained; RTOEAA = Resistance training overtrained with essential amino acid supplementation; Plantaris (A); Soleus (B); No significant difference was found between the different groups. Plantaris- F = 1.482, P = 0.241; Soleus- F = 2.88, P = 0.0691.
