# Supplementary material for: The Effect of an Essential Amino Acid Supplement on Muscle Performance and Inflammation in an Animal Model of Overtraining
Source: Nutrients. 2026 Aug 10;18(16):2614. doi: 10.3390/nu18162614 (PMC13516721; doi:10.3390/nu18162614)

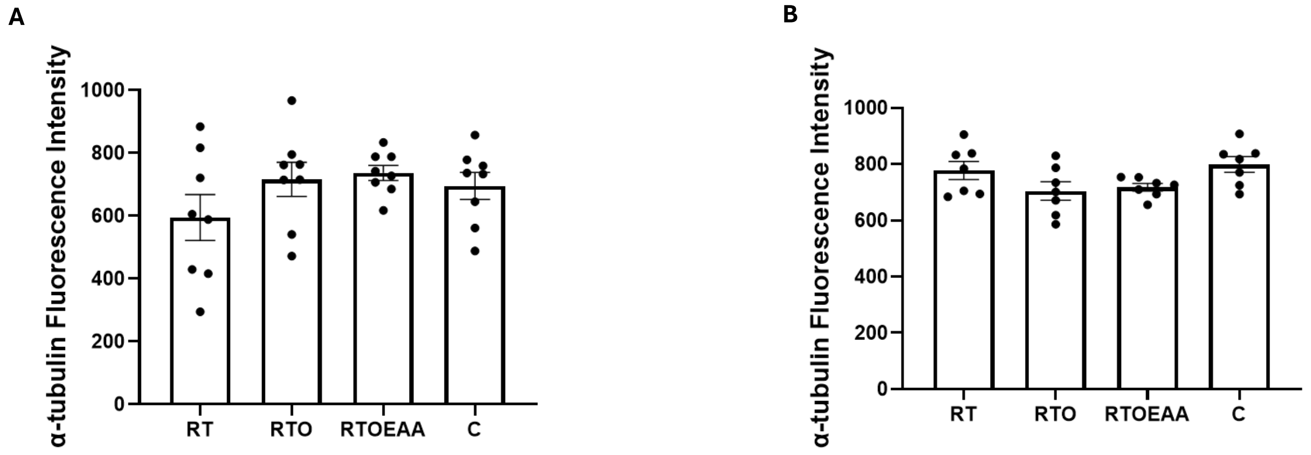

Supplementary Figure S1. α-tubulin Fluorescence Intensity. RT = Resistance training; RTO = Resistance training overtrained; RTOEAA = Resistance training overtrained with essential
amino acid supplementation; Plantaris (A); Soleus (B); No significant difference was found between the different groups. Plantaris- $F = 1.482$, $P = 0.241$; Soleus- $F = 2.88$, $P = 0.0691$.

AR Full Blot

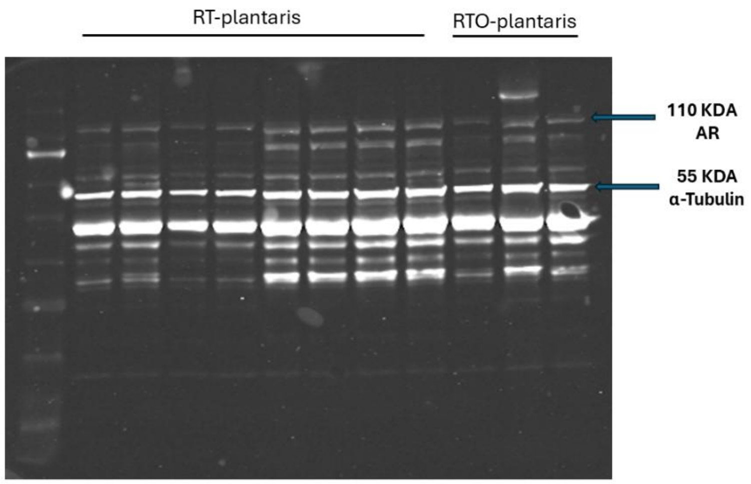

AR Full Blot

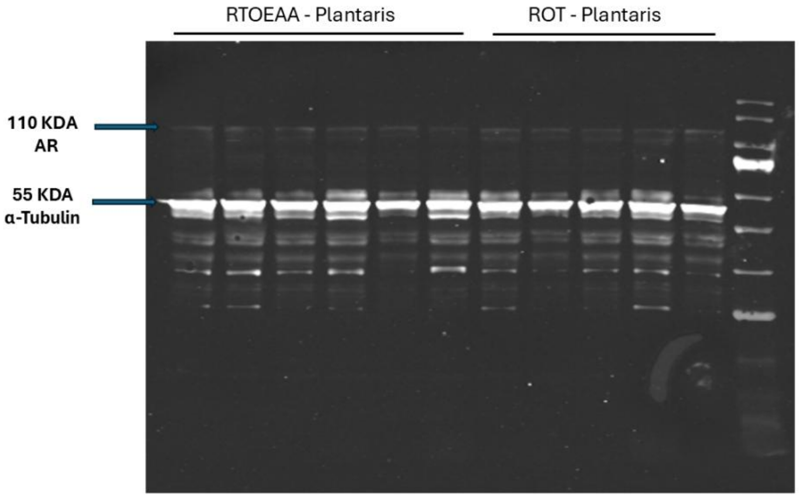

AR Full Blot

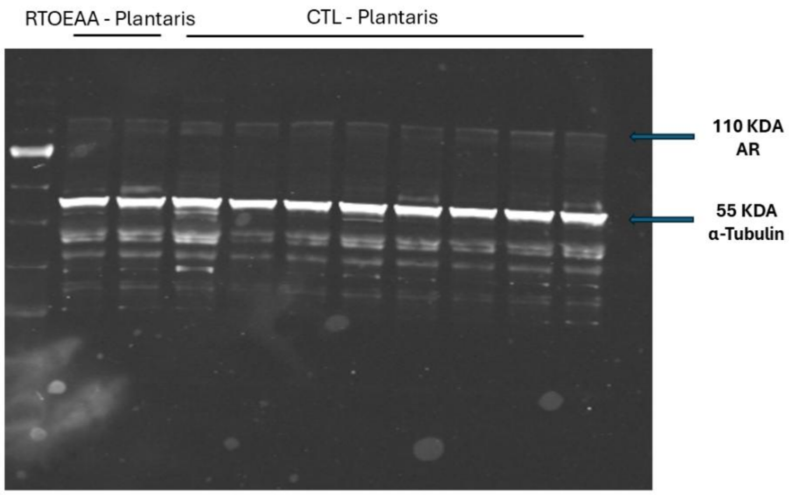

AR Full Blot

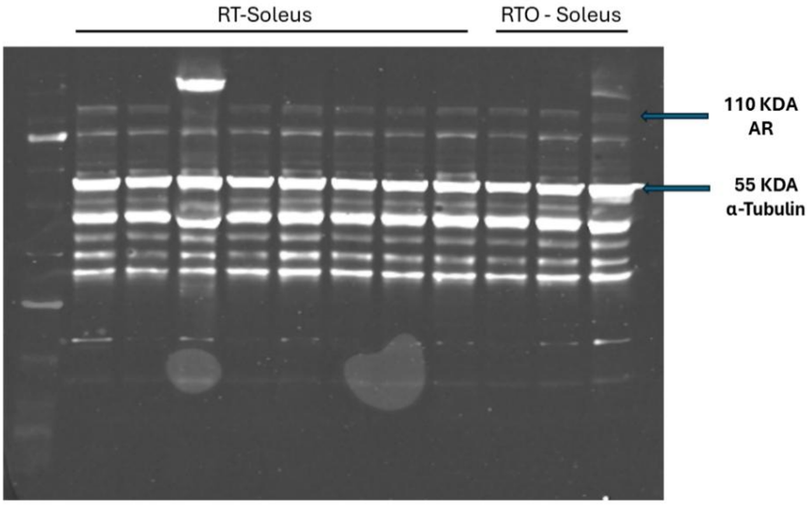

AR Full Blot

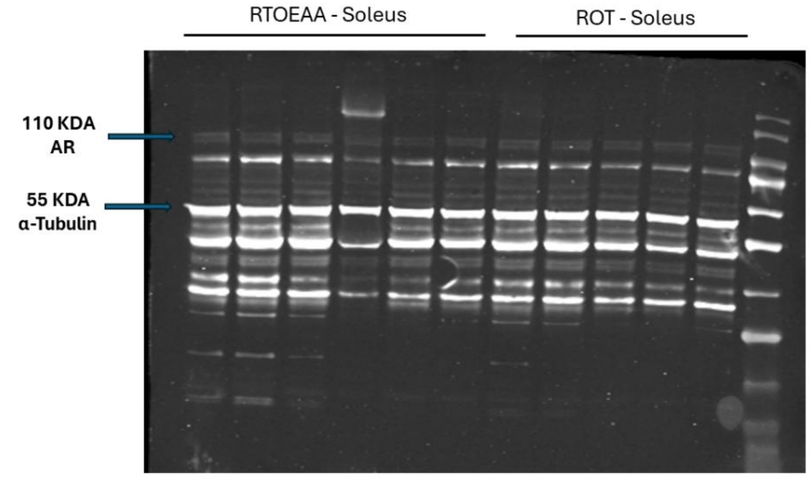

AR Full Blot

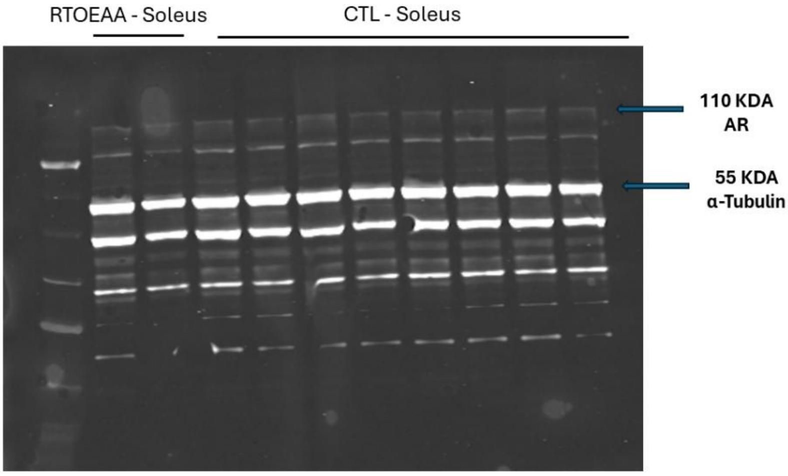

GR Full Blot

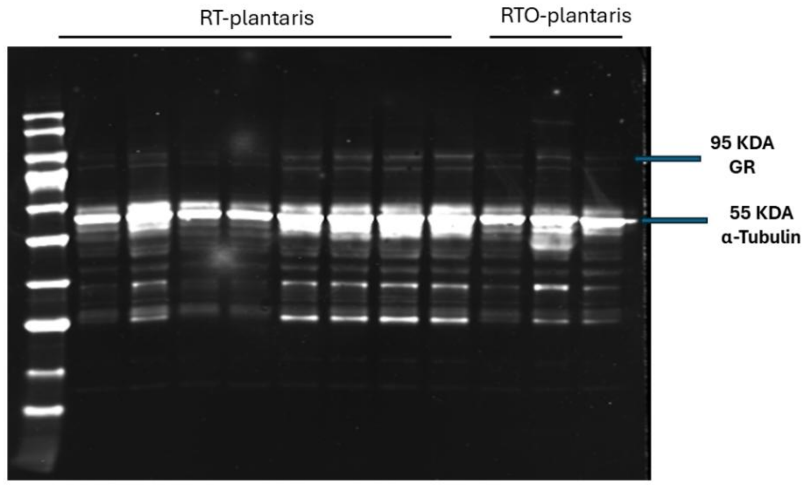

GR Full Blot

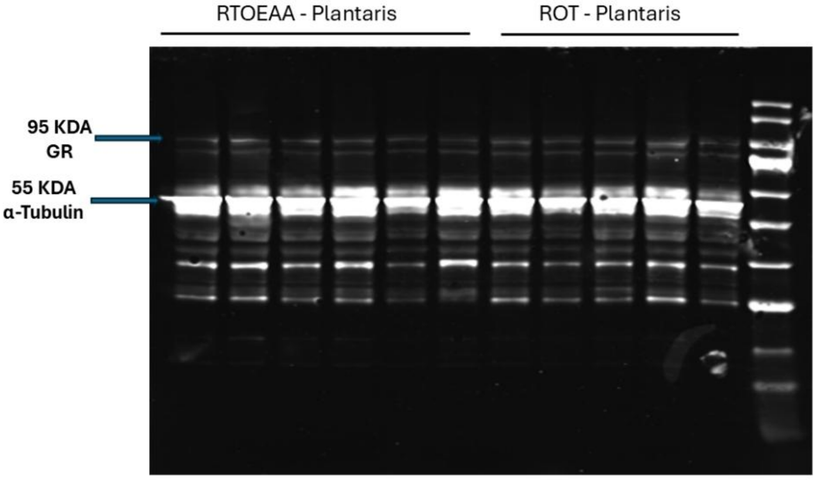

GR Full Blot

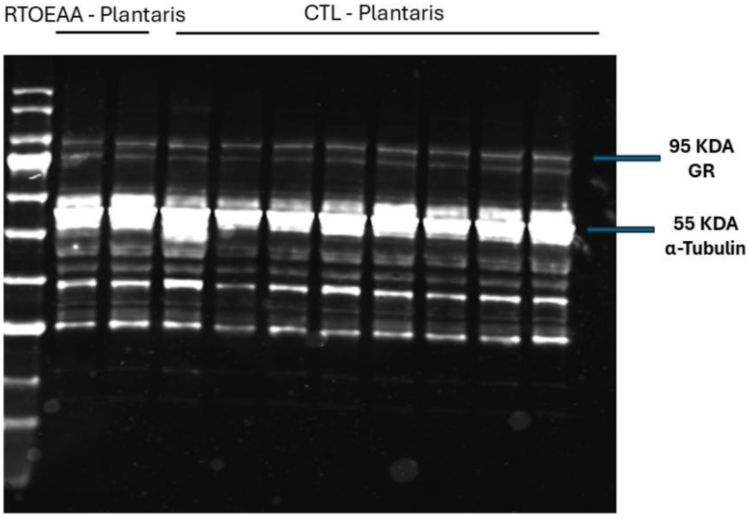

GR Full Blot

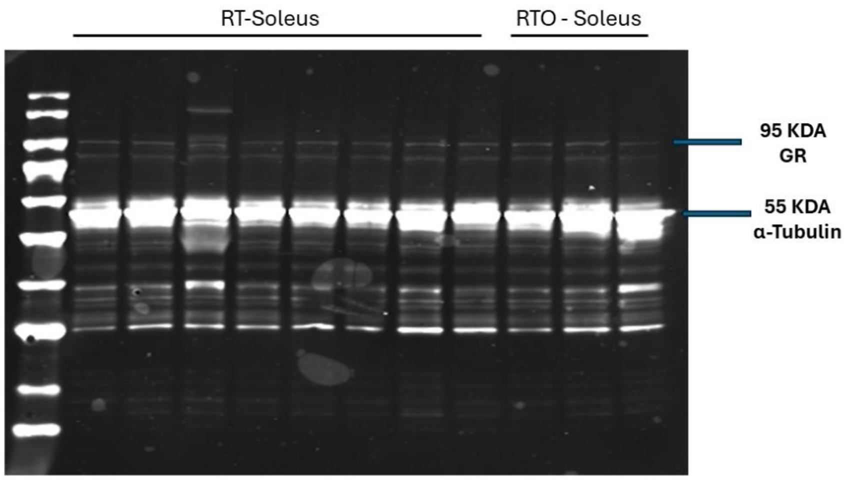

GR Full Blot

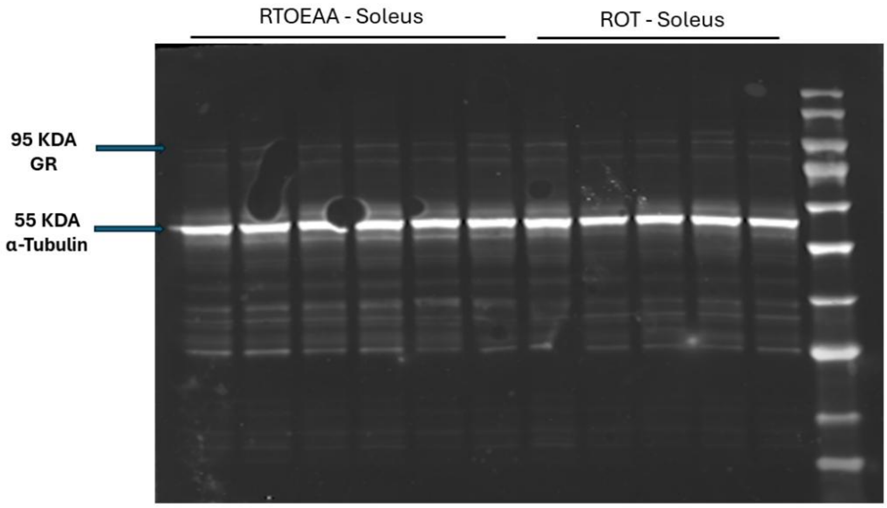

GR Full Blot

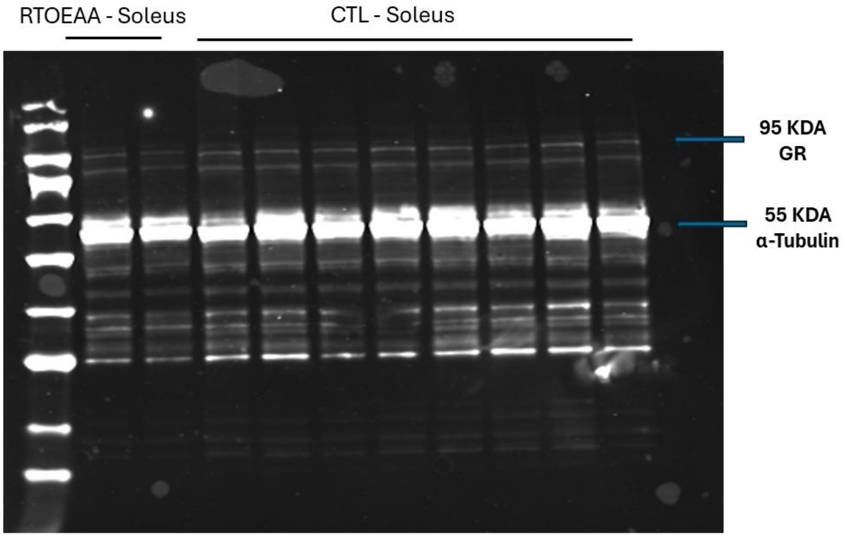

TNF-α Full Blot

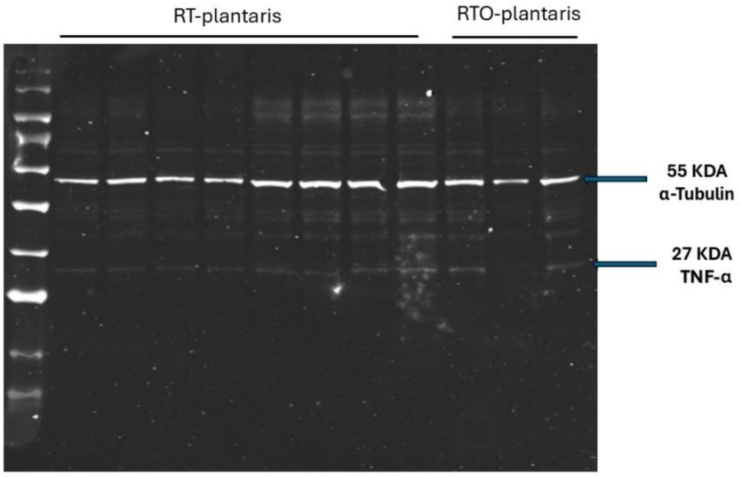

TNF-α Full Blot

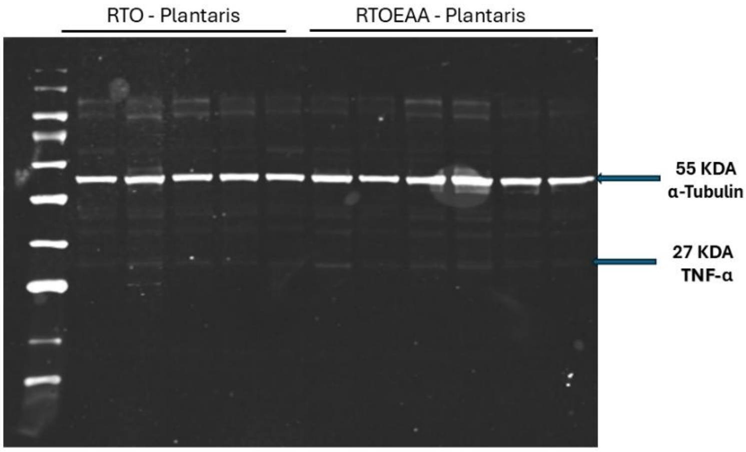

TNF-α Full Blot

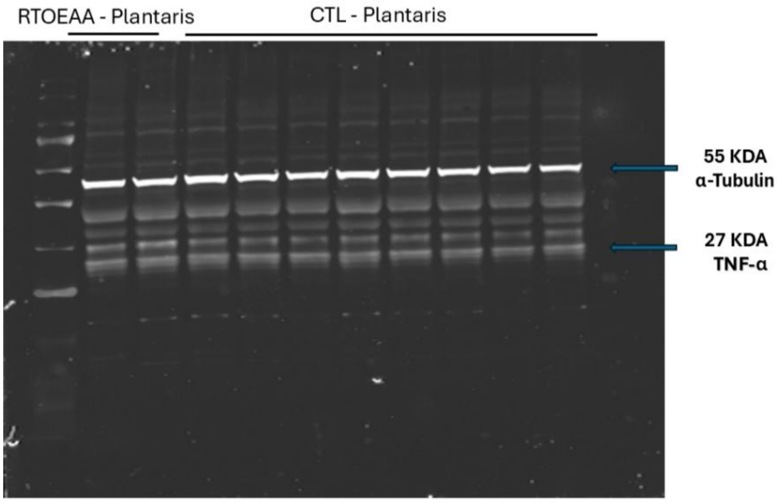

TNF-α Full Blot

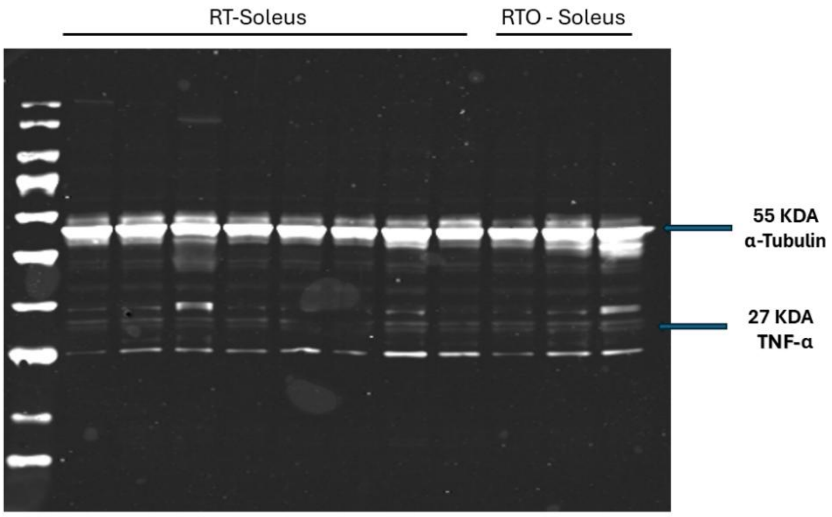

TNF-α Full Blot

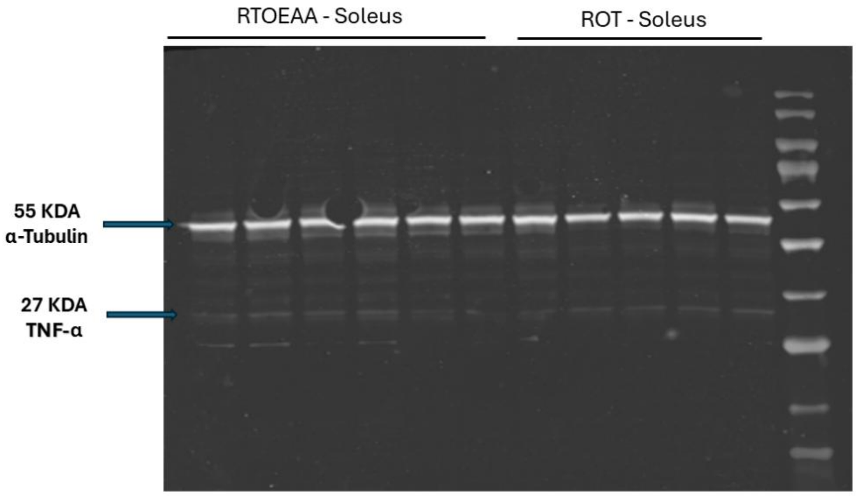

TNF-α Full Blot

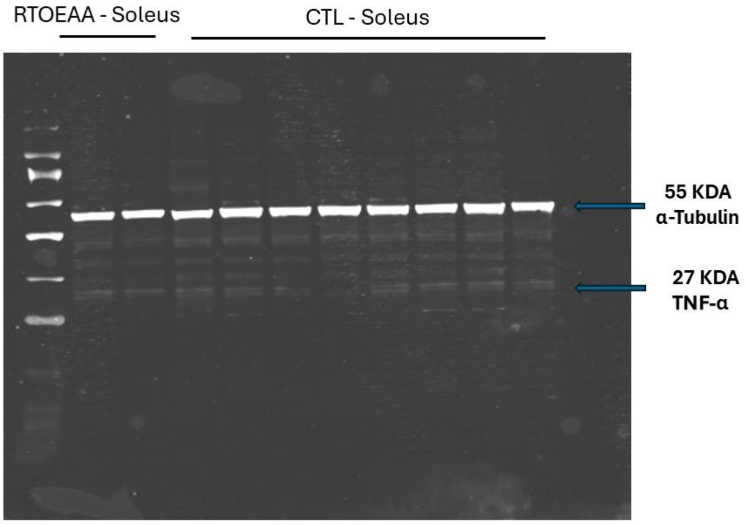

Supplement: Supplementary file 1 [file nutrients-18-02614-s001.zip › nutrients-4472224-supplementary.pdf]
